# The impact of COVID-19 pandemic on invasive fungal infections in Africa: What have we learned?

**DOI:** 10.1371/journal.pntd.0010720

**Published:** 2022-08-30

**Authors:** Chibuike Ibe

**Affiliations:** Departments of Microbiology, Faculty of Biological Sciences, Abia State University, Uturu, Nigeria; Universidad de Antioquia, COLOMBIA

## Abstract

Invasive fungal infections (IFIs) have been described as diseases of the poor. The mortality rate of the infections is comparable to that of malaria, HIV, and TB, yet the infections remain poorly funded, neglected in research, and policy at all levels of human resources. The Coronavirus Disease 2019 (COVID-19) pandemic has further worsened the current state of management for IFIs. At the same time, response to COVID-19 has stirred and boosted vaccine production, vaccine substance manufacturing, and building of next-generation sequencing capacity and genomics data sharing network in the continent. Through collaboration and transdisciplinary research effort, these network and technology can be extended to encourage fungal research to address health issues of existing and emerging fungal pathogens.

## Africa: Current statistics

Africa, especially sub-Saharan Africa, has unique characteristics that particularly define health outcomes and emergency response in the region (see S1 Table) [1]. According to the United Nations, there is an estimated 7.7 billion people on the planet, and Africa makes up around one-sixth. This figure is projected to reach 9.7 billion by 2050, and sub-Saharan Africa alone with an estimated population of more than a billion is expected to double. Nine countries including 5 of the most populated in Africa: Ethiopia, Democratic Republic of Congo, Tanzania, Egypt, and Nigeria are expected to show the highest growth during this time. Increasing population has consequences in health, and Africa has very low density of healthcare workforce with the average healthy life expectancy of 54.8 years in sub-Saharan Africa. Health expenditure in Africa is low and most countries do not have universal health coverage, which has been the reason for catastrophic out-of-pocket coverage for healthcare expenses by mostly the populace in rural communities. Moreover, one-third of sub-Saharan Africa population live in extreme poverty, while more than half the population requires intervention against neglected tropical diseases (NTDs).

### State of IFIs and COVID-19

Invasive fungal infections (IFIs) including fungal NTDs (FNTDs) such as mycetoma, paracoccidioidomycosis, and chromoblastomycosis and AIDS-associated fungal infection such as cryptococcosis, candidosis, aspergillosis, and histoplasmosis are endemic in sub-Saharan Africa and other parts of the world with the above characteristics. IFIs have been typically characterised as infections of the poor and have been the target of African governments and organisations such as World Health Organisation (WHO), Global Action Fund for Fungal Infection (GAFFI), and Leading International Fungal Education (LIFE). However, the development of rapid and accurate diagnostic tools and availability of better therapeutic options for the management of the complex medical presentations of IFIs have been deemed less important than that of other infections including malaria, HIV, and tuberculosis (TB). The morbidity and rate of mortality of IFIs are comparable to that of these infections, but IFIs continue to be neglected in research, funding, policy, and human resources at all levels. Fungal infections remain underappreciated and the least prioritised (see S1 Table).

The Coronavirus Disease 2019 (COVID-19) pandemic caused by Severe Acute Respiratory Syndrome Coronavirus 2 (SARS-CoV-2) has devastated the healthcare system of African countries and the world. The infection is characterised by flu-like symptoms, breathing difficulty, and sore throat; was first reported in Wuhan, China; and has since infected more than 11.3 million people and caused 251,666 deaths in Africa in about 2 years [2], and it is arguably the most important healthcare challenge of the 21st century. According to John Nkengasong, the Director of Africa Centre for Disease Control and Prevention (Africa CDC), the pandemic has left African countries woefully short in healthcare responses [3]. Data from Zambia suggested that the impact of COVID-19 in Africa is significantly underestimated [4]. There is no doubt that this emerging infection has affected the established healthcare and public health structures for known health issues including IFIs and FNTDs; the question is, have we learned anything?

### Did COVID-19 impact IFIs management?

**Fig 1:**
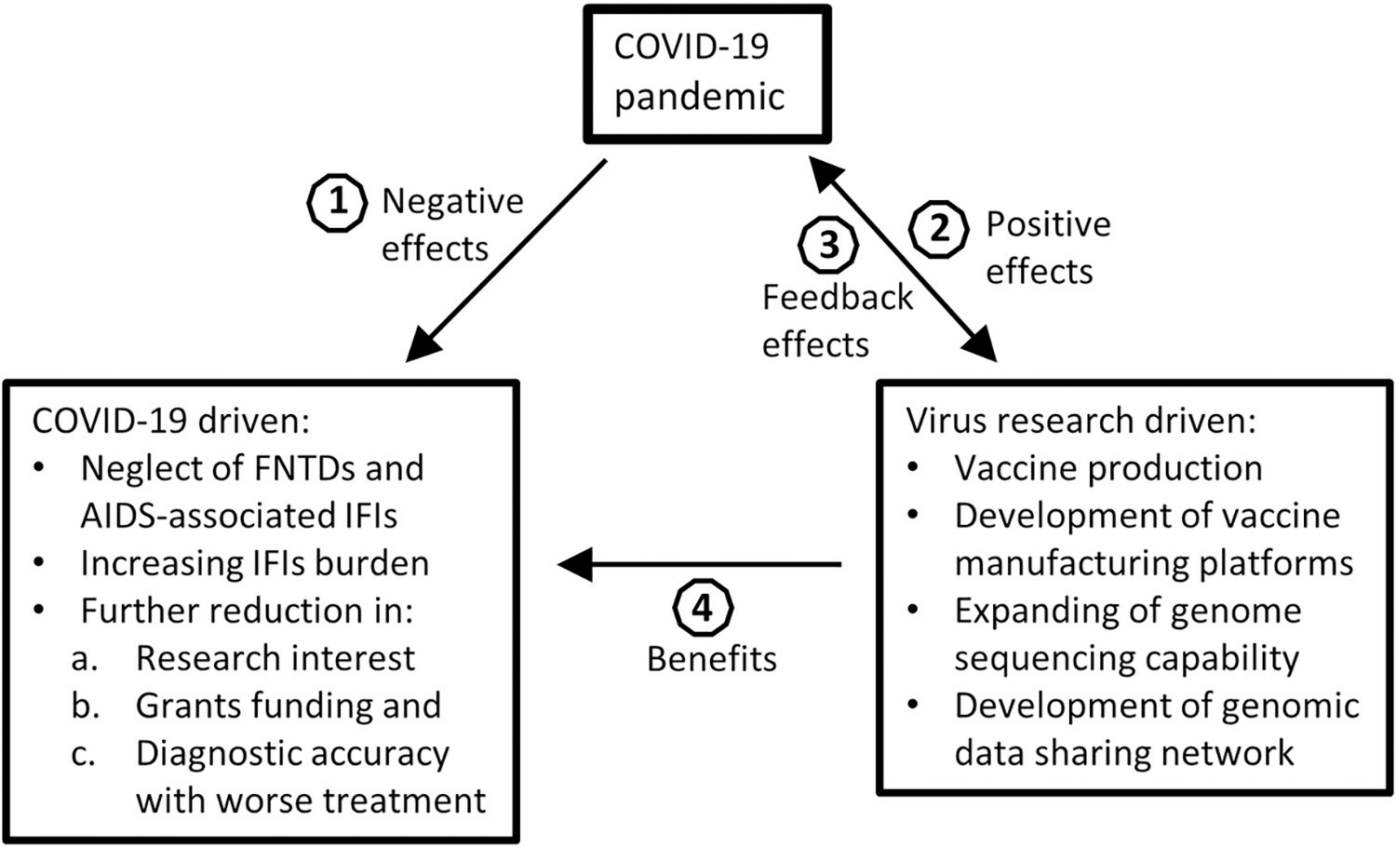
Lessons learned from COVID-19 pandemic influence on IFIs. In endemic regions, IFIs, malaria, HIV, and TB are common due to poverty. However, due to limited awareness, and low research funding, limited rapid and accurate diagnostics, and effective therapeutic options, compared to malaria, HIV, and TB, IFIs have not received adequate priority despite their high prevalence. (1) Negative effects of COVID-19 on IFIs: The emergence of COVID-19 pandemic attracted all medical attention, resources and funding for emergency response and containment, and research; thereby, it is assumed to exacerbate the existing conditions of IFIs and increase burden. (2) Positive effects of COVID-19 response: Through sheer ambition and strategic investment including contributions from donors such as Illumina, Oxford Nanopore, Microsoft, and Gates Foundation, African governments have pledged to expand vaccine production and strengthen vaccine substance manufacturing capacity to meet with up to 60% of local vaccine needs in the next 2 decades. The response to COVID-19 also stimulated expansion of capacity and expertise in next-generation sequencing technology and deepened collaboration in genomic data sharing networks through African Pathogen Genomics Initiative. (3) Feedback effects: These technologies and networks are now used in surveillance of SARS-CoV-2 evolution to tackle new threats. (4) Benefits: The virus-driven development in genome sequencing and vaccine manufacturing technologies and genomic data sharing network have wide application and can be used to drive research in IFIs and other neglected infections through collaborative efforts. COVID-19, Coronavirus Disease 2019; FNTD, fungal neglected tropical disease; IFI, invasive fungal infection; SARS-CoV-2, Severe Acute Respiratory Syndrome Coronavirus 2; TB, tuberculosis.

IFIs are reported mainly in the regions with described characteristics. Though there have been schemes aimed at screening to mitigate fungal infections, such as the CDC/CHAI/Unitaid initiative towards ending cryptococcosis deaths by 2030, the allocation of more funds for the diagnosis and treatment of malaria, HIV, and TB has made fungal infections remain a major concern (see S1 Table). Furthermore, fungal infections are always secondary, have relatively long latency period, and being that superficial nonfatal infections are common, IFIs are misconceived, affecting awareness among the endemic areas’ dwellers, and even among clinicians [5]. Fungal infections are also significantly influenced by other infections in so many ways (Fig 1). In reports detailing grant funding for infectious diseases research, fungal infections research had the least grant allocation [6,7]. There are no records of grant funding allocation for FNTDs. However, research in cryptococcosis seem to be the only relatively funded compared to various other fungal infections. A recent survey of 40 hospitals across African countries showed that diagnostic tests for *Cryptococcus* species were available in more hospitals than tests for other fungal pathogens [8]. The survey further showed that first line therapies for treating IFIs were limited.

COVID-19 global challenge may have different outcomes on fungal infections (Fig 1). This will include the suppression of T-cell immunity, which usually promotes IFIs especially cryptococcosis. More broadly, in IFIs superinfection with COVID-19, COVID-19 will likely take priority in diagnosis and treatment due to its rapid life-threatening manifestations. Fungal infections, though also life threatening, often show delayed manifestation due to the slow growing nature of fungal pathogens. Additionally, as more research is geared towards understanding the infection pathogenesis, developing rapid and accurate diagnostics, and treatment options and better prevention and control measures for COVID-19, more grants are awarded to tackle the infection globally. Thus, grant funding for fungal pathogens research will be low (if not diverted to COVID-19 response), especially in Africa where the pathogens are a major health problem, affecting the gains made in the control of their infections. Malaria, HIV, and TB have had serious negative effects on the outcome of IFIs over the years [6], and COVID-19 is unlikely to improve the situation (Fig 1). For example, invasive pulmonary aspergillosis (IPA) and invasive candidosis affect mainly immunocompromised patients with high mortality rates. IPA prevalence is as high as 23% in ICU patients with severe influenza. IPA and invasive candidosis with COVID-19 superinfection have been reported in patients with acute respiratory distress syndrome (ARDS) [9,10]. The prevalence of IPA with COVID-19 superinfection is as high as 33% with peak mortality rate of 60% [9]. However, accurately diagnosing IPA in a COVID-19 superinfection is a challenge still. The prevalence and mortality rate of COVID-19 with pulmonary candidosis superinfection in patients in Egypt have been reported to be very high [10]. Pulmonary candidosis superinfection in COVID-19 patients with ARDS has been ventilator acquired [10]. COVID-19 patients with mucormycosis superinfection have also been reported at 6.7% with 91% mortality in Egypt [11]. Data on COVID-19 and IFIs superinfection are limited, and the effect of COVID-19 on IFIs healthcare structure as well as the socioeconomic impact is undefined. Thus, the true picture of COVID-19 impact remains unclear.

### What have we learned or gained from COVID-19 pandemic response? Can they be used in IFIs research?

Countering COVID-19 pandemic is one of the biggest challenges facing the healthcare systems and researchers all over the world. However, the health consequences of COVID-19 with IFIs superinfection have not been well understood. In resource-poor settings, COVID-19 patients are at a high risk of developing candidosis, *Pneumocytis* pneumonia, mould infection/pulmonary aspergillosis, and mucormycosis [12,13]. This risk is significantly associated with prolonged use of mechanical ventilator, underlying diabetic conditions, corticosteroid use, and damage to pulmonary epithelial cells due to severe influenza [10,11]. Data emanating from Egypt suggested high morbidity and mortality in cases of COVID-19 and candidosis or mucormycosis superinfection. IFIs in COVID-19 patients continues to be a problem partly because of the waves of infection with new variants of SARS-CoV-2. So far, first-generation COVID-19 vaccines effective in preventing infection with the wild-type SARS-CoV-2 do not confer immunity against the emerging SARS-CoV-2 variants, and cases of COVID-19 infection are currently on the rise again.

The COVID-19 pandemic has evolved beyond healthcare challenges to rising societal concerns. In Africa, this was a wake-up call. African countries were at the backend of nations waiting to receive and roll out COVID-19 vaccination for their citizens, while high-income countries have received more than 87% of the global vaccine stock [14]. This inequity gap has caused countries and continents to rethink their healthcare investment and the pace of infrastructural development. In Africa, it is now clear that less than 1% of the vaccines used in the continent is made by manufacturers in 5 countries: South Africa, Egypt, Morocco, Tunisia, and Senegal [3,15] (see S1 Table). However, on 13 April 2021, African governments committed to an ambitious plan of boosting vaccine production to 60% in the next 20 years through bolstering research and development and building the required infrastructures and facilities. Africa CDC plans to set up 5 more vaccine production centres in Africa and invest about $400 million through the African Development Bank to fund 2 new vaccine technology platforms for vaccine production [3]. This will ultimately help build vaccine production capacity in Africa.

Vaccine production in Africa will require heavy long-term funding commitment to expanded research capacity and build strategic regulatory guidance and support bodies and stakeholders and government commitment to purchase the produced vaccines [3]. The establishment of African continental free trade area in 2021 guarantees a supply network that will aid uptake of the vaccines by the 1.3 billion Africans.

Transdisciplinary research efforts can help tackle the healthcare and societal concerns raised by the COVID-19 pandemic, and through collaborative efforts, fungal pathogens research can benefit from the SARS-CoV-2-driven technological innovations and development (Fig 1). The vaccine technology platforms and vaccine substance manufacturing technology already in place in Tunisia, Senegal, and Egypt (see S1 Table) can stimulate fungal vaccine research. Whole-genome sequencing technology and networks available, which have been driven by viral research to provide real-time data on the mutation and evolution of viral pathogens [16], also have the potential to improve fungal vaccine and antifungal targets research in Africa. Data from Democratic Republic of Congo on Ebola virus response and research have shown that a combination of genomic and epidemiological surveillance can be used in response to infectious disease outbreak and containment [17]. The African Pathogen Genomics Initiative network, which links genomic sequencing laboratories in Africa, holds great promise in fungal genomics research and surveillance of emerging infections such as emergomycosis and mucormycosis, data sharing, and can improve fungal genome sequencing capability across the continent. Through collaborative research effort, this network and the technologies are available to fungal researchers while also positioning Africa for a better response for future viral or fungal pandemic.

## Conclusions

The population and health characteristics of the African continent may explain why some infections are endemic in the region. The emergence of COVID-19 has made worse existing health conditions and the management of IFIs but at the same time has encouraged the strengthening of health institutions and training of experts to withstand future shock and respond to outbreaks, respectively.

Novel strategies for the management of the COVID-19 pandemic and transdisciplinary research that includes researchers from all disciplines ranging from life sciences/biomedical sciences to health sciences to physical sciences and engineering to social sciences and economics working independently or as a unit will be crucial in managing health implications of the pandemic [18]. This effort will emphasise shared conceptual frameworks that integrate discipline-specific methods [19].

Interestingly, the transdisciplinary frameworks, vaccine production technologies, and infrastructures built in response to the COVID-19 pandemic can find application in fungal pathogens research including in antifungals and diagnostics research. As fungal infections thrive in T-cell immunosuppression associated with viral infections, collaborative research between virologists and mycologists in this era of virus research–driven high-end technological evolution holds a lot of promise in improving African welfare and beyond.

## Supporting information

S1 Table The population and some characteristics of African countries. (DOCX)
