# Supplementary material for: The impact of COVID-19 pandemic on invasive fungal infections in Africa: What have we learned?
Source: PLoS Negl Trop Dis. 2022 Aug 30;16(8):e0010720. doi: 10.1371/journal.pntd.0010720 (PMC9426908; doi:10.1371/journal.pntd.0010720)
Supplement: S1 Table — (DOCX) [file pntd.0010720.s001.docx]

Supplementary information

**S1 Table: The population and some characteristics of African countries**

| **Country** | **Population size^1^** | **Health expenditure (% GDP) (2019)^3^** | **Out-of-pocket expenditure (% health expenditure) (2019)^3^** | **Density per 10 000 population (2011-2019)^2^** | | **Population that needs interventions against NTDs (2019)^2^** | **Healthy life expectancy at birth (yr) (2019)^2^** | **Total burden of mycoses** | **Serious mycoses** | **Vaccine manufacturing capacity^4^** |
| --- | --- | --- | --- | --- | --- | --- | --- | --- | --- | --- |
|  |  |  |  | **Nurse** | **Doctor** |  |  |  |  |  |
| Nigeria | 206 139 589 | 3.0 | 70.5 | 15.0 | 3.8 | 134 545 208 | 54.4 | 17 983 517 ^1^ | 492 340 ^1^ | PVSM |
| Ethiopia | 114 963 588 | 3.2 | 37.8 | 7.1 | 0.8 | 76 238 251 | 59.9 | 8 820 437 ^2^ | 118 319 ^2^ | PVSM |
| Egypt | 102 334 404 | 4.7 | 62.7 | 19.3 | 7.5 | 6 894 411 | 63.0 | 1 649 686 ^3^ | 327 420 ^3^ | VSM and fill and finish |
| DR Congo | 89 561 403 | 3.5 | 38.5 | 11.1 | 0.9 | 53 320 501 | 54.1 | 5 177 427 ^4^ | 72 936* ^4^ |  |
| Tanzania | 59 734 218 | 3.8 | 22.1 | 5.8 | 0.6 | 27 086 592 | 58.5 | 1 422 204 | 73 144 ^5^ |  |
| South Africa | 59 308 690 | 9.1 | 5.6 | 13.1 | 7.9 | 18 807 465 | 56.2 | 4 047 138 | 266 991 ^6^ | Only fill and finish  PVSM |
| Kenya | 53 771 296 | 4.5 | 24.3 | 11.7 | 1.6 | 8 321 398 | 57.7 | 3 186 523 ^7^ | 459 187 ^7^ |  |
| Uganda | 45 741 007 | 3.8 | 38.2 | 12.4 | 1.7 | 24 639 995 | 58.2 | 2 500 000 ^8^ | 781 439 ^8^ |  |
| Sudan | 43 849 260 | 4.5 | 67.3 | 11.5 | 2.6 | 12 015 065 | 59.9 | - | - |  |
| Algeria | 43 851 044 | 6.2 | 33.4 | 15.5 | 17.2 | 10 339 | 66.4 | 568 942 ^9^ | 78 657 ^9^ |  |
| Morocco | 36 910 560 | 5.3 | 46.8 | 13.9 | 7.3 | 5 576 | 63.7 | - | - |  |
| Angola | 32 866 272 | 2.5 | 37.4 | 4.1 | 2.1 | 15 362 008 | 54.8 | - | - |  |
| [Mozambique](https://worldpopulationreview.com/countries/mozambique-population) | 31 255 435 | 7.8 | 9.9 | 4.7 | 0.8 | 21 517 399 | 50.4 | 1 836 374 ^10^ | 46 484 ^10^ |  |
| [Ghana](https://worldpopulationreview.com/countries/ghana-population) | 31 072 940 | 3.4 | 36.2 | 27.1 | 1.1 | 17 220 101 | 58.0 | 1 030 563 ^11^ | 30 000 ^11^ | PVSM |
| [Madagascar](https://worldpopulationreview.com/countries/madagascar-population) | 27 691 018 | 3.6 | 32.5 | 3.0 | 1.8 | 21 125 732 | 57.3 | 1 178 319 ^12^ | 7 069* ^12^ |  |
| [Cameroon](https://worldpopulationreview.com/countries/cameroon-population) | 26 545 863 | 3.6 | 72.5 | 0.1 | 0.9 | 16 891 418 | 54.5 | 1 126 332 ^13^ | 45 477 ^13^ |  |
| [Ivory Coast](https://worldpopulationreview.com/countries/ivory-coast-population) | 26 378 274 | 3.3 | 37.2 | 6.6 | 1.6 | 21 713 670 | 54.8 | 1 744 277 ^14^ | 16 521* ^14^ |  |
| [Niger](https://worldpopulationreview.com/countries/niger-population) | 25 847 386 | 5.6 | 46.1 | 2.2 | 0.4 | 14 046 246 | 55.5 | - | - |  |
| [Burkina Faso](https://worldpopulationreview.com/countries/burkina-faso-population) | 20 903 273 | 5.4 | 34.6 | 9.3 | 0.9 | 3 652 080 | 54.9 | 1 360 280 ^15^ | 16 747 ^15^ |  |
| [Mali](https://worldpopulationreview.com/countries/mali-population) | 20 250 833 | 3.8 | 31.3 | 4.4 | 1.3 | 7 735 946 | 54.6 | - | - |  |
| [Malawi](https://worldpopulationreview.com/countries/malawi-population) | 19 129 952 | 7.3 | 16.8 | 4.4 | 0.4 | 12 393 153 | 57.1 | 1 338 523 ^16^ | 49 837 ^16^ |  |
| [Zambia](https://worldpopulationreview.com/countries/zambia-population) | 18 383 955 | 5.3 | 10.2 | 10.2 | 0.9 | 12 032 435 | 54.4 | 897 421 ^17^ | 26 960* ^17^ |  |
| [Senegal](https://worldpopulationreview.com/countries/senegal-population) | 16 743 927 | 4.1 | 50.9 | 5.4 | 0.9 | 8 815 619 | 59.4 | 1 743 507 ^18^ | 26 633 ^18^ | VSM and fill and finish |
| [Chad](https://worldpopulationreview.com/countries/chad-population) | 16 425 864 | 4.3 | 57.2 | 1.4 | 0.5 | 6 270 047 | 52.0 | - | - |  |
| [Somalia](https://worldpopulationreview.com/countries/somalia-population) | 15 893 222 | - | - | 1.1 | 0.2 | 2 286 299 | 49.7 | - | - |  |
| [Zimbabwe](https://worldpopulationreview.com/countries/zimbabwe-population) | 14 862 924 | 7.7 | 24.3 | 19.3 | 2.1 | 10 660 813 | 53.1 | 2 212 715 ^19^ | 24 945* ^19^ |  |
| [Guinea](https://worldpopulationreview.com/countries/guinea-population) | 13 132 795 | 3.9 | 59.2 | 1.2 | 0.8 | 7 480 197 | 53.3 |  | - |  |
| [Rwanda](https://worldpopulationreview.com/countries/rwanda-population) | 12 952 218 | 6.4 | 11.6 | 9.5 | 1.2 | 5 015 979 | 60.2 | - | - |  |
| [Benin](https://worldpopulationreview.com/countries/benin-population) | 12 123 200 | 2.3 | 47.0 | 3.0 | 0.6 | 6 070 676 | 55.5 | - | - |  |
| [Burundi](https://worldpopulationreview.com/countries/burundi-population) | 11 890 784 | 7.9 | 24.6 | 6.6 | 1.0 | 3 418 124 | 55.6 | - | - |  |
| [Tunisia](https://worldpopulationreview.com/countries/tunisia-population) | 11 818 619 | 6.9 | 37.9 | 25.1 | 13.0 | 7 085 | 66.9 | - | - | VSM and fill and finish |
| [South Sudan](https://worldpopulationreview.com/countries/south-sudan-population) | 11 193 725 | 6.0 | 23.5 | - | - | 8 472 338 | 53.7 | - | - |  |
| [Togo](https://worldpopulationreview.com/countries/togo-population) | 8 278 724 | 5.7 | 66.2 | 4.6 | 0.8 | 4 311 460 | 56.2 | 384 404 ^20^ | 4 159* ^20^ |  |
| [Sierra Leone](https://worldpopulationreview.com/countries/sierra-leone-population) | 7 976 983 | 8.7 | 55.1 | 7.5 | 0.7 | 6 910 107 | 52.9 | 376,643 ^21^ | 7 834* ^21^ |  |
| [Libya](https://worldpopulationreview.com/countries/libya-population) | 6 871 292 | 6.0 | 36.6 | 65.3 | 20.9 | 6 774 | 65.2 | - | - |  |
| Congo | 5 518 087 | 2.0 | 45.8 | 9.3 | 1.1 | 1 407 153 | 56.2 | 293 918 ^22^ | 5 552* ^22^ |  |
| [Liberia](https://worldpopulationreview.com/countries/liberia-population) | 5 057 681 | 8.4 | 54.4 | 5.3 | 0.4 | 3 175 460 | 54.9 | - | - |  |
| [Central African Republic](https://worldpopulationreview.com/countries/central-african-republic-population) | 4 829 767 | 7.7 | 60.3 | 2.1 | 0.7 | 4 442 825 |  | - |  |  |
| [Mauritania](https://worldpopulationreview.com/countries/mauritania-population) | 4 649 658 | 3.3 | 45.0 | 9.3 | 1.9 | 826 827 | 59.8 | - | - |  |
| [Eritrea](https://worldpopulationreview.com/countries/eritrea-population) | 3 546 421 | 4.4 | 43.2 | 14.4 | - | 427 112 | 55.7 | - | - |  |
| [Namibia](https://worldpopulationreview.com/countries/namibia-population) | 2 540 905 | 8.5 | 8.2 | 19.5 | 5.9 | 1 094 020 | 56.1 | 60,456 ^23^ | 6,545 ^23^ |  |
| [Gambia](https://worldpopulationreview.com/countries/gambia-population) | 2 416 668 | 3.8 | 23.1 | 6.1 | 1.1 | 168 211 | 57.0 | - | - |  |
| [Botswana](https://worldpopulationreview.com/countries/botswana-population) | 2 351 627 | 6.0 | 3.1 | 54.6 | 2.9 | 238 203 | 53.9 | - | - |  |
| [Gabon](https://worldpopulationreview.com/countries/gabon-population) | 2 225 734 | 2.7 | 23.0 | 29.5 | 6.8 | 937 923 | 57.6 | - | - |  |
| [Lesotho](https://worldpopulationreview.com/countries/lesotho-population) | 2 142 249 | 11.2 | 13.7 | 32.6 |  | 382 336 | 44.2 | - | - |  |
| [Guinea Bissau](https://worldpopulationreview.com/countries/guinea--bissau-population) | 1 968 001 | 8.3 | 65.1 | 6.9 | 1.3 | 1 232 549 | 52.6 | - | - |  |
| [Equatorial Guinea](https://worldpopulationreview.com/countries/equatorial-guinea-population) | 1 402 985 | 3.1 | 75.4 | 5.0 | 4.0 | 429 326 | 53.9 | - | - |  |
| [Mauritius](https://worldpopulationreview.com/countries/mauritius-population) | 1 271 768 | 6.2 | 42.7 | 35.2 | 25.3 | 0 | 63.9 | - | - |  |
| [Eswatini](https://worldpopulationreview.com/countries/eswatini-population) | 1 160 164 | 6.7 | 10.5 | 41.4 | 1,0 | 406 184 | 50.1 | - | - |  |
| [Djibouti](https://worldpopulationreview.com/countries/djibouti-population) | 988 000 | 1.8 |  | 2.2 | 7.3 | 110 561 | 58.0 | - | - |  |
| Comoros | 869,601 | 5.1 | 61.8 | 6.3 | 1.7 | 788 813 | 58.9 |  |  |  |
| [Cape Verde](https://worldpopulationreview.com/countries/cape-verde-population) | 555 987 | 4.8 | 24.9 | 13.0 | 7.8 | 137 073 | 64.8 | - | - |  |
| [Sao Tome and Principe](https://worldpopulationreview.com/countries/sao-tome-and-principe-population) | 219 159 | 5.5 | 17.6 | 3.2 | 19.2 | 201 114 | 61.6 | - | - |  |
| [Seychelles](https://worldpopulationreview.com/countries/seychelles-population) | 98 347 | 5.1 | 25.2 | 98.5 | 24.7 | 0 | 64.0 | - | - |  |
|  | **North sum = 245 635 179**  **Sub-Sahara sum = 1 079 950 945**  **Total sum = 1 342 467 346** | **North mean = 5.6**  **Sub-Sahara mean = 5.3**  **Total mean = 5.2** | **North mean = 47.5**  **Sub-Sahara mean = 36.2**  **Total mean = 37.5** | **North mean = 25.1**  **Sub-Sahara mean = 13.4**  **Total mean = 14.3** | **North mean = 11.4**  **Sub-Sahara mean = 3.3**  **Total mean = 4.3** | **North sum = 18 939 250**  **Sub-Sahara sum = 590 370 087**  **Total sum = 611 706 197** | **North mean = 64.2**  **Sub-Sahara mean = 54.8**  **Total mean = 55.8** | **Total sum = 60 939 606** | **Total sum = 2 985 196** |  |

NTDs, Neglected tropical diseases; yr, year; vaccine substance manufacturing; planned vaccine substance manufacturing

^1^ www.worldpopulationreview.com

^2^www.who.int

^3^www.worldbank.org

^4^Irwin A: https://www.nature.com/articles/d41586-021-01048-1

*Estimated from systemic invasive, deep-seated, and potentially fatal infections

**Reference**

1. Oladele, R. O. & Denning, D. W. Burden of serious fungal infection in Nigeria. West African Journal of Medicine. 2014;**33**:107–114.

2. Tufa, T. B. & Denning, D. W. The burden of fungal infections in Ethiopia. Journal of Fungi. 2019;**5**:1–14.

3. Zaki, S. M. & Denning, D. W. Serious fungal infections in Egypt. *European Journal of Clinical* Microbiology and Infectious Diseases. 2017;**36**:971–974.

4. Kamwiziku, G. K., Makangara, J. C. C., Orefuwa, E. & Denning, D. W. Serious fungal diseases in Democratic Republic of Congo – Incidence and prevalence estimates. Mycoses. 2021;64:1159–1169.

5. Faini, D. *et al.* Burden of serious fungal infections in Tanzania. Mycoses.2015;**58**:70–79.

6. Schwartz, I. S. *et al.* The estimated burden of fungal disease in South Africa. South African Medical Journal**. 2019;109:**885.

7. Guto, J. A., Bii, C. C. & Denning, D. W. Estimated burden of fungal infections in Kenya. Journal of Infection in Developing Countries. 2016;**10**,:777–784.

8. Parkes-Ratanshi, R. *et al.* Cryptococcal disease and the burden of other fungal diseases in Uganda; Where are the knowledge gaps and how can we fill them? Mycoses. 2015;**58**:85–93 .

9. Chekiri-Talbi, M. & Denning, D. W. Burden of fungal infections in Algeria. *European Journal of* Clinical Microbiology and Infectious Diseases. 2017;**36**:999–1004.

10. Sacarlal, J. & Denning, D. W. Estimated burden of serious fungal infections in Mozambique. Journal of Fungi**. 2018;4:**1–11.

11. Ocansey, BK. *et al.* Estimated Burden of Serious Fungal Infections in Jamaica. Journal of Fung**. 2019;5**,:1–15.

12. Rakotoarivelo, R. A. Razanamparany, V. R. & Rakotomizao, Jocelyn Denning, D. W. The burden of serious fungal disease in Madagascar. in 7th Congress on Trends in Medical Mycology. 2015;1.

13. Mandengue, C. E. & Denning, D. W. The burden of serious fungal infections in Cameroon. Journal of Fungi**.** 2018**;4:** 1–9.

14. Koffi, D. *et al.* Estimates of serious fungal infection burden in Côte d’Ivoire and country health profile. Journal of Medical Mycology. 2018;1–8 (2020) doi:10.1016/j.mycmed.2020.101086.

15. Bamba, S. *et al.* Burden of severe fungal infections in Burkina Faso. Journal of Fungi**. 2018;4:** 1–9.

16. Kalua, K., Zimba, B. & Denning, D. W. Estimated burden of serious fungal infections in Malawi. Journal of Fungi**.** 2018**;4:**1–7.

17. Chishimba, L., Niven, M. & Denning, W. Burden of serious fungal infections in Zambia. in *6th* Trends in Medical Mycology*.* 2014.

18. Badiane, A. S., Ndiaye, D. & Denning, D. W. Burden of fungal infections in Senegal. Mycoses. 2015;**58:**63–69.

19. Pfavayi, L. T., Denning, D. W., Baker, S., Sibanda, E. N. & Mutapi, F. Determining the burden of fungal infections in Zimbabwe. Scientific Reports**.** 2011;**11**;1–13.

20. Dorkenoo, A. M. *et al.* Estimated burden of serious fungal infections in Togo. Mycoses**. 2021;64**:1535–1541.

21. Lakoh, S. *et al.* The burden of serious fungal infections in Sierra Leone: a national estimate. Therapeutic Advances in Infectious Disease. 2021;8.

22. Amona, F. M., Denning, D. W., Moukassa, D. & Hennequin, C. Current burden of serious fungal infections in Republic of Congo. Mycoses. 2020;**63**:543–552.

23. Dunaiski, C. M. & Denning, D. W. Estimated burden of fungal infections in Namibia. Journal of Fungi. 2019;**5**:1–15.
